# Molecular hydrogen suppresses activated Wnt/β-catenin signaling

**DOI:** 10.1038/srep31986

**Published:** 2016-08-25

**Authors:** Yingni Lin, Bisei Ohkawara, Mikako Ito, Nobuaki Misawa, Kentaro Miyamoto, Yasuhiko Takegami, Akio Masuda, Shinya Toyokuni, Kinji Ohno

**Affiliations:** 1 Division of Neurogenetics, Center for Neurological Diseases and Cancer, Nagoya University Graduate School of Medicine, Nagoya, Japan; 2 Department of Pathology and Biological Responses, Graduate school of Medicine, Nagoya University Graduate School of Medicine, Nagoya, Japan

## Abstract

Molecular hydrogen (H_2_) is effective for many diseases. However, molecular bases of H_2_ have not been fully elucidated. Cumulative evidence indicates that H_2_ acts as a gaseous signal modulator. We found that H_2_ suppresses activated Wnt/β-catenin signaling by promoting phosphorylation and degradation οf β-catenin. Either complete inhibition of GSK3 or mutations at CK1- and GSK3-phosphorylation sites of β-catenin abolished the suppressive effect of H_2_. H_2_ did not increase GSK3-mediated phosphorylation of glycogen synthase, indicating that H_2_ has no direct effect on GSK3 itself. Knock-down of adenomatous polyposis coli (APC) or Axin1, which form the β-catenin degradation complex, minimized the suppressive effect of H_2_ on β-catenin accumulation. Accordingly, the effect of H_2_ requires CK1/GSK3-phosphorylation sites of β-catenin, as well as the β-catenin degradation complex comprised of CK1, GSK3, APC, and Axin1. We additionally found that H_2_ reduces the activation of Wnt/β-catenin signaling in human osteoarthritis chondrocytes. Oral intake of H_2_ water tended to ameliorate cartilage degradation in a surgery-induced rat osteoarthritis model through attenuating β-catenin accumulation. We first demonstrate that H_2_ suppresses abnormally activated Wnt/β-catenin signaling, which accounts for the protective roles of H_2_ in a fraction of diseases.

The effects of H_2_ have been reported in 166 disease models and human diseases^1^. Prominent effects have been observed especially in oxidative stress-mediated diseases and inflammatory diseases. H_2_ was first reported to be a selective scavenger of ^•^OH and peroxynitrite^2^. Cumulative evidence, however, suggests that H_2_ functions as a signaling modulator^3^,^4^,^5^. In this study, we dissected the effects of H_2_ on Wnt/β-catenin signaling.

Wnt/β-catenin signaling controls cell proliferation and differentiation by regulating expression of target genes. In the absence of Wnt ligands, β-catenin is steadily phosphorylated by casein kinase 1 (CK1) at Ser45 and glycogen synthase kinase 3 (GSK3) at Ser33/Ser37/Thr41 at its N-terminus in a degradation complex assembled by Axin1 and adenomatous polyposis coli (APC), and is subsequently degraded through the β-transducin repeat-containing protein (β-TrCP)-mediated ubiquitin/proteasome pathway^6^. Wnt ligands or GSK3 inhibitors [lithium chloride (LiCl) and 6-bromoindirubin-3′-oxime (BIO)] suppress phosphorylation and degradation of β-catenin. Consequently, β-catenin accumulates in the cytoplasm and then translocates into the nucleus to interact with T-cell factor/lymphoid enhancing factor (TCF/LEF) to activate transcription of the Wnt/β-catenin target genes.

Aberrant activation of Wnt/β-catenin signaling is associated with a number of diseases including cancers and degenerative diseases^7^. Osteoarthritis (OA) is characterized by degradation of extracellular matrix (ECM) molecules, loss of articular cartilages, and formation of osteophytes. Development and aggravation of OA are associated with abnormal activation of Wnt/β-catenin signaling^8^,^9^,^10^. H_2_ is beneficial for musculoskeletal diseases including inflammatory and mitochondrial myopathies^11^, microgravity-induced bone loss^12^, post-ovariectomy osteopenia^13^, rheumatoid arthritis (RA)^14^,^15^, and psoriasis-associated arthritis^16^. However, no study has demonstrated the effect of H_2_ on OA to the best of our knowledge.

In this study, we observed that H_2_ inhibited Wnt/β-catenin signaling activated by Wnt3a, LiCl, or BIO in L and HeLa cells. H_2_ promoted phosphorylation, ubiquitination, and subsequent degradation of β-catenin without directly affecting mRNA level of β-catenin. The effect of H_2_ required CK1/GSK3-phosphorylation sites on β-catenin, the CK1/GSK3 activities, as well as APC and Axin1 activities. We confirmed the suppressive effect of H_2_ on Wnt/β-catenin signaling in chondrocytes and observed a protective effect of H_2_ on OA progression. We report that H_2_ is an inhibitor for activated Wnt/β-catenin signaling, which provides additional evidence that H_2_ is a gaseous signal modulator.

## Results

### H_2_ suppresses activated Wnt/β-catenin signaling

**Figure 1:**
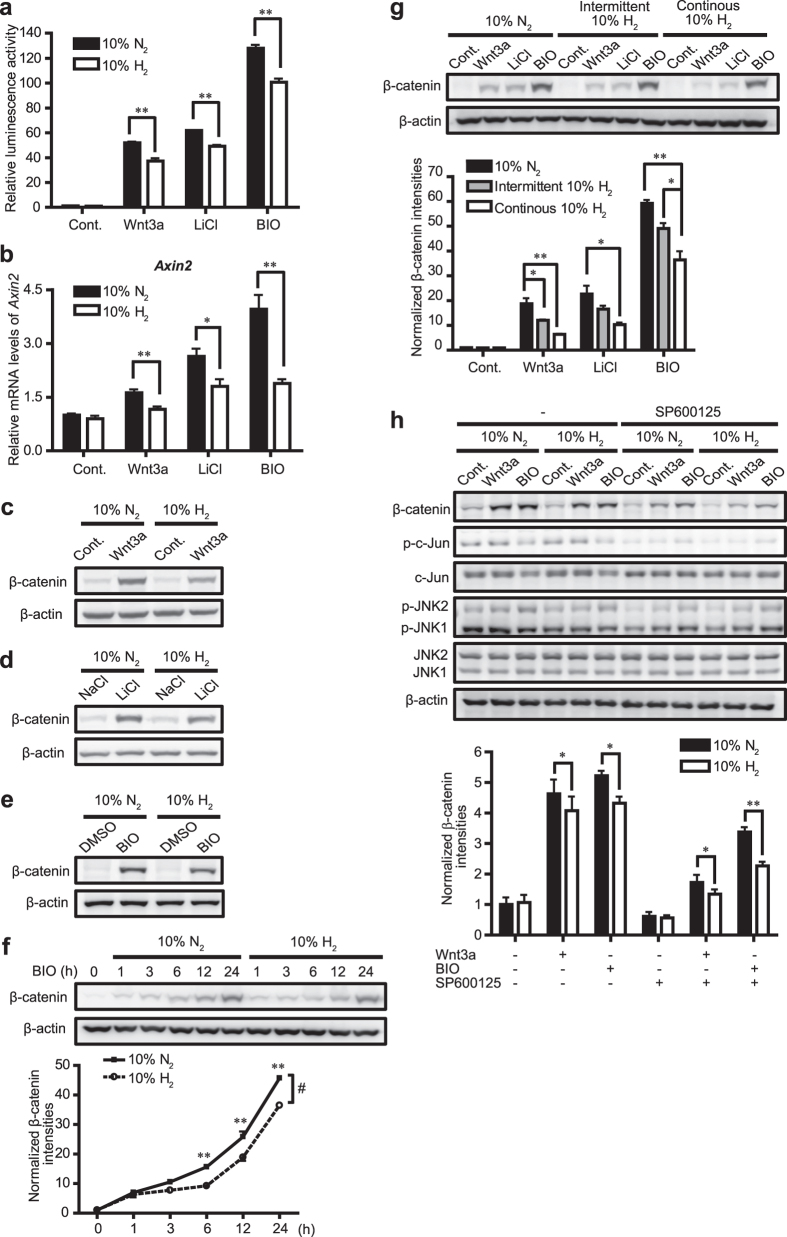
H_2_ suppresses Wnt/β-catenin signaling in L cells. **(a,b)** Cells were treated with control CM (Cont.), Wnt3a CM, 30 mM LiCl, or 2 μM BIO with 10% H_2_ or 10% N_2_ gas for 24 h. The Wnt/β-catenin signaling activity was measured by Topflash luciferase reporter assay (*n* = 4) **(a)** and expression of *Axin2* was quantified by qRT-PCR (*n* = 3–4) **(b)**. *P < 0.05 and **P < 0.01 by Student’s *t*-test. **(c–e)** Cells were treated with pairs of CM (Cont.) and Wnt3a CM **(c)**; 30 mM NaCl and 30 mM LiCl **(d)**; and 0.02% DMSO and 2 μM BIO/0.02% DMSO **(e)** with 10% H_2_ or 10% N_2_ gas for 24 h. Representative Western blots are shown. **(f)** Cells were treated with 2 μM BIO with 10% H_2_ or 10% N_2_ gas for indicated periods of time. The upper panel shows representative Western blots and the lower panel shows densitometry of β-catenin/β-actin (*n* = 4). ^#^*P* < 0.05 by two-way repeated measures ANOVA. **P < 0.01 by Student’s *t*-test with Bonferroni correction for each pair of H_2_ and N_2_. **(g)** Cells were treated with control CM (Cont.), Wnt3a CM, 30 mM LiCl, or 2 μM BIO with either 10% N_2_ gas, intermittent 10% H_2_ gas, or continuous 10% H_2_ gas for 24 h. Representative Western blots are shown with densitometry of β-catenin/β-actin (*n* = 3). *P < 0.05 and **P < 0.01 by Student’s *t*-test with Bonferroni correction. **(h)** Cells were pretreated with 40 μM SP600125 for 30 min. Cells were then added with control CM (Cont.), Wnt3a CM, or 2 μM BIO with 10% H_2_ or 10% N_2_ gas for 1 h. Representative Western blots are shown with densitometry of β-catenin/β-actin (*n* = 4). *P < 0.05 and **P < 0.01 by Student’s *t*-test.

In order to examine whether H_2_ affects Wnt/β-catenin signaling, we first conducted Topflash luciferase reporter assay in L cells with 10% H_2_ or 10% nitrogen (N_2_) gas. Topflash luciferase reporter plasmid carries 8 copies of TCF-binding sites in the promoter region and the firefly luciferase cDNA to quantify activation of Wnt/β-catenin signaling. Addition of Wnt3a or a GSK3 inhibitor, LiCl or BIO, to the culture medium for 24 h increased Topflash reporter activity, which, however, was attenuated by H_2_ (Fig. 1a). Similar effects of H_2_ on the Wnt/β-catenin signaling were also observed in HeLa cells (Supplementary Fig. S1a), suggesting that H_2_ suppressed activation of Wnt/β-catenin signaling in different cell lines. We also examined the expression of an endogenous target gene of Wnt/β-catenin signaling, *Axin2*, and found the suppressive effect of H_2_ on Wnt3a-, LiCl-, or BIO-induced upregulation of *Axin2* mRNA in L cells (Fig. 1b). Then, we examined whether H_2_ decreases the level of β-catenin, the transcriptional co-activator, by Western blotting. H_2_ reduced accumulation of endogenous β-catenin induced by Wnt3a, LiCl, or BIO (Fig. 1c–e), as well as accumulation of exogenous myc-β-catenin (Supplementary Fig. S1c) in L cells. Consistently, the nuclear accumulation of β-catenin induced by Wnt3a, LiCl, or BIO was also decreased by H_2_ in L cells (Supplementary Fig. S1d). H_2_, however, failed to suppress basal expression level of β-catenin in HeLa cells (Supplementary Fig. S1e). Time course analysis revealed that the suppressive effect of H_2_ on β-catenin accumulation was prominent in the first 6 h in L cells (Fig. 1f and supplementary Fig. S1f). In all the experiments stated above, we used 10% N_2_ gas as a control for 10% H_2_ gas to match O_2_ concentrations. We observed that minimal reduction of O_2_ concentration by 10% N_2_ gas had no effect on the β-catenin level (Supplementary Fig. S1g). Therefore, we consistently used 10% N_2_ gas in a control group in the following experiments.

We previously reported a prominent protective effect of administration of H_2_ water and intermittent inhalation of H_2_ gas, but not continuous inhalation of H_2_ gas, in a rat model of Parkinson’s disease^17^. Therefore, we examined whether intermittent exposure to H_2_ gas has a more suppressive effect on Wnt/β-catenin signaling. We then followed the protocol of intermittent administration of H_2_ gas as described previously^17^. Briefly, L cells were exposed to 10% H_2_ gas for 15 min followed by air for 45 min using a time controller, and the cycle was repeated for 24 h. We added 5% CO_2_ throughout the cycle. We found that intermittent H_2_ treatment was less effective than continuous H_2_ treatment on suppressing β-catenin level (Fig. 1g).

H_2_ has been reported to inhibit mitogen-activated protein (MAP) kinase signaling in cell lines and rodent disease models^3^,^4^,^5^. A previous report shows that inhibition of JNK, but not of ERK or p38 MAP kinase, decreases Wnt3a-induced β-catenin accumulation^18^. As we found that H_2_ suppressed Wnt/β-catenin signaling, we next asked whether H_2_ suppresses Wnt/β-catenin signaling through inhibition of JNK signaling. We pretreated L cells with 40 μM SP600125, which efficiently inhibited JNK-mediated phosphorylation of c-Jun (Fig. 1h). L cells were then added with Wnt3a or BIO with 10% H_2_ or 10% N_2_ gas for 1 h. SP600125 decreased Wnt3a- or BIO-induced β-catenin accumulation, but did not abolish the suppressive effect of H_2_ on β-catenin accumulation (Fig. 1h), suggesting that the effect of H_2_ on Wnt/β-catenin signaling is independent of JNK signaling.

Then, we asked whether the suppressive effect of H_2_ on Wnt/β-catenin signaling is operational *in vivo*. A previous study shows that starvation induces nuclear accumulation of β-catenin in the liver in mice^19^. Consistently, we detected accumulation of β-catenin in the liver of starved mice. *Ad libitum* oral intake of H_2_ water suppressed β-catenin accumulation in the liver in starved but not fed mice (Supplementary Fig. S1h). All these data point to the notion that H_2_ suppresses activation of Wnt/β-catenin signaling by reducing β-catenin accumulation.

### H_2_ promotes β-catenin degradation

**Figure 2:**
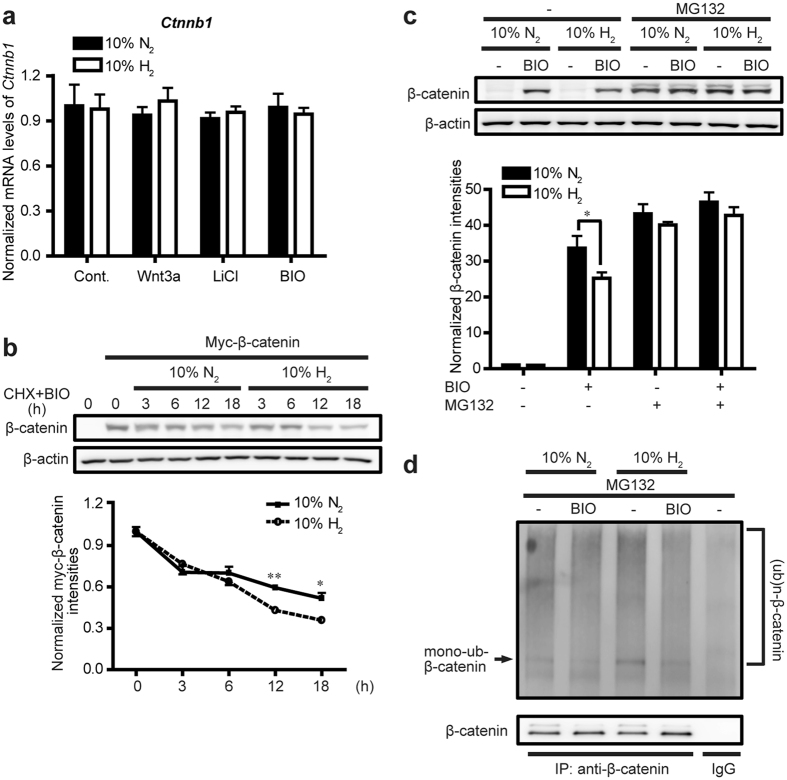
H_2_ promotes β-catenin degradation in L cells. **(a)** Cells were treated with control CM (Cont.), Wnt3a CM, 30 mM LiCl, or 2 μM BIO with 10% H_2_ or 10% N_2_ gas for 24 h. Expression of *Ctnnb1* encoding β-catenin was quantified by qRT-PCR (*n* = 3). **(b)** Cells transfected with myc-β-catenin (XE28 XBC plasmid) were exposed to a combination of 10 μg/ml cycloheximide (CHX) and 2 μM BIO with 10% H_2_ or 10% N_2_ gas for indicated periods of time. Representative Western blots are shown with densitometry of myc-β-catenin/β-actin (*n* = 4). Two groups were not statistically different by two-way repeated measures ANOVA. *P < 0.05 and **P < 0.01 by Student’s *t*-test with Bonferroni correction for each pair of H_2_ and N_2_. **(c)** Cells were treated with 2 μM BIO, 10 μM MG132, or a combination of both with 10% H_2_ or 10% N_2_ gas for 12 h. Representative Western blots are shown with densitometry of β-catenin/β-actin (*n* = 6). Note that mono-ubiquitinated β-catenin is visible in MG132-treated cells. *P < 0.05 by Student’s *t*-test. **(d)** Cells were treated with 10 μM MG132 in the presence or absence of 2 μM BIO with 10% H_2_ or 10% N_2_ gas for 12 h. Cells were then immunoprecipitated (IP) with antibodies against β-catenin or mouse control IgG (IgG). Blots were immunostained with antibodies against ubiquitin and β-catenin, and representative blots are shown.

We found that mRNA levels of β-catenin remained unchanged by H_2_ treatment in the absence and presence of Wnt/β-catenin signaling activators (Wnt3a, BIO, or LiCl) in L cells (Fig. 2a) and HeLa cells (Supplementary Fig. S2a), indicating that H_2_ acts on β-catenin protein synthesis/degradation rather than its gene expression. To further dissect this hypothesis, we used cycloheximide (CHX) to block protein biosynthesis of β-catenin and conducted the CHX chase assay to determine whether H_2_ accelerates the degradation rate of β-catenin. Because endogenous β-catenin is hard to be detected in L cells, we expressed myc-β-catenin in L cells and found that H_2_ accelerated degradation of myc-β-catenin (Fig. 2b). We additionally performed the CHX chase assay in HeLa cells and found that H_2_ modestly accelerated degradation of endogenous β-catenin (Supplementary Fig. S2b,c).

Intracellular β-catenin is degraded by the ubiquitin-proteasome system. Consequently, we used MG132 for proteasome inhibition to determine whether the ubiquitin–proteasome system is involved in H_2_-induced down-regulation of β-catenin. We found that MG132 minimized the suppressive effect of H_2_ on BIO-induced β-catenin accumulation (Fig. 2c). We also observed that H_2_ facilitated ubiquitination of β-catenin (Fig. 2d). These results suggest that H_2_ enhances proteasome-mediated β-catenin degradation.

### H_2_ enhances β-catenin phosphorylation

**Figure 3:**
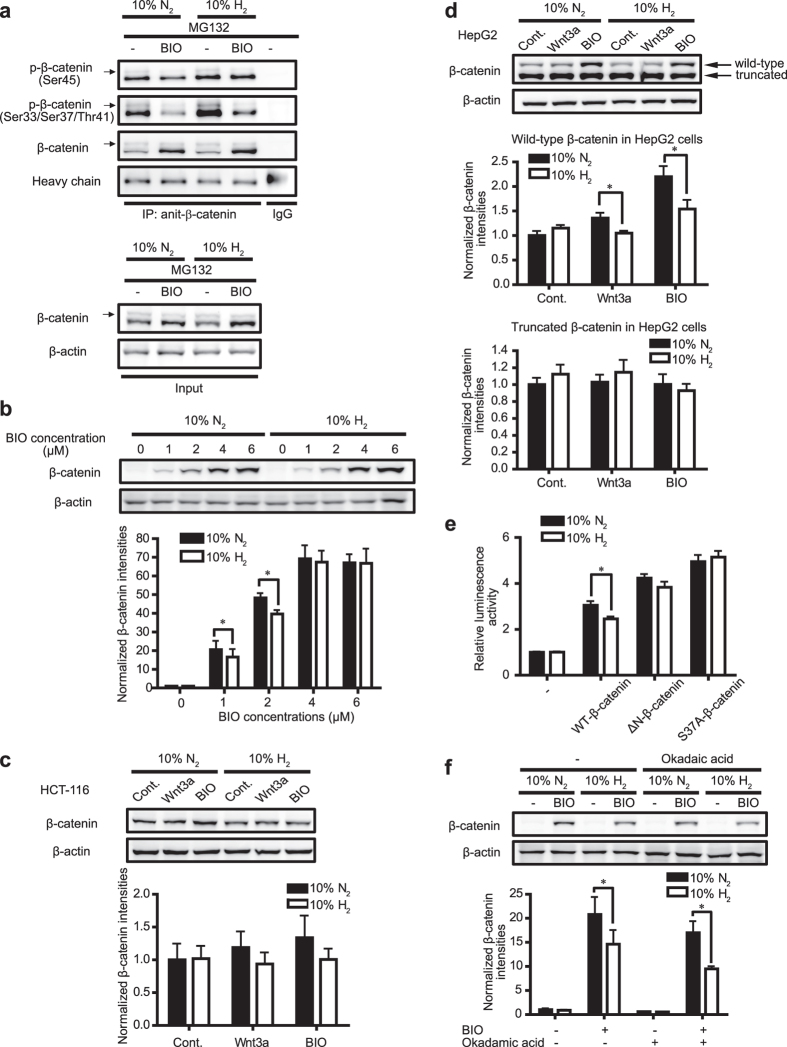
H_2_ facilitates β-catenin phosphorylation. **(a)** L cells were treated with 10 μM MG132 in the presence or absence of 2 μM BIO with 10% H_2_ or 10% N_2_ gas for 6 h. Cell lysates were then immunoprecipitated (IP) with an antibody against β-catenin or mouse control IgG (IgG). Blots were immunostained with an antibody against phospho-β-catenin (at Ser45), phospho-β-catenin (at Ser33/Ser37/Thr41) and β-catenin. Representative Western blots of IP samples and input samples were shown in the upper panels and lower panels, respectively. Arrows indicate mono-ubiquitinated phospho-β-catenin. **(b)** L cells were exposed to indicated concentrations of BIO with 10% H_2_ or 10% N_2_ gas for 24 h. Representative Western blots are shown with densitometry of β-catenin/β-actin (*n* = 4). *P < 0.05 by Student’s *t*-test with Bonferroni correction. **(c,d)** HCT-116 cells **(c)** and HepG2 cells **(d)** were incubated with control CM (Cont.), Wnt3a CM, or 2 μM BIO with 10% H_2_ or 10% N_2_ for 24 h. HCT-116 cells carry Ser45-deleted β-catenin in one allele. HepG2 cells carry truncated β-catenin in one allele. Representative Western blots are shown with densitometry of β-catenin/β-actin (*n* = 3). Wild-type and Ser45-deleted β-catenin were analyzed together for HCT-116 cells **(c)**, whereas wild-type and truncated β-catenin were individually analyzed for HepG2 cells **(d)**. *P < 0.05 by Student’s *t*-test. **(e)** L cells were transfected with wild-type (HA-WT-β-catenin) or mutant forms of HA-β-catenin (HA-ΔN-β-catenin and HA-S37A-β-catenin). Cell were then treated with 10% H_2_ or 10% N_2_ gas for 24 h. Wnt/β-catenin signaling activity was measured by Topflash luciferase reporter assay (*n* = 4). *P < 0.05 by Student’s *t*-test. **(f)** L cells were pretreated with 30 nM okadaic acid for 30 min. Cells were then added with 2 μM BIO with 10% H_2_ or 10% N_2_ gas for 12 h. Representative Western blots are shown with densitometry of β-catenin/β-actin (*n* = 4). *P < 0.05 by Student’s *t*-test.

In canonical Wnt/β-catenin signaling, β-catenin is sequentially phosphorylated at Ser45 by CK1 and at Ser33/Ser37/Thr41 by GSK3 in a complex with Axin1 and APC. Phosphorylation of these residues is required to trigger proteasome-mediated β-catenin degradation^20^. We then tested whether the effect of H_2_ was on either phosphorylation or degradation of β-catenin. Since the level of phospho-β-catenin is dependent on the total amount of β-catenin, we added MG132 to block β-catenin degradation in the proteasome pathway. H_2_ increased phosphorylation of β-catenin at Ser45 and at Ser33/Ser37/Thr41 with the treatment of MG132 (Fig. 3a). The concentration of BIO (2 μM) used in these studies was likely to partially inhibit GSK3 activity in L cells, because the phosphorylations on Ser33/Ser37/Thr41 of β-catenin were detected to some extent even in the presence of 2 μM BIO (Fig. 3a). We thus increased BIO concentrations to examine whether the effect of H_2_ requires GSK3 activity. We found that H_2_ was able to decrease β-catenin accumulation induced by low BIO concentrations (1 μM and 2 μM) but not by high BIO concentrations (4 μM and 6 μM) (Fig. 3b). These results indicate that the inhibitory effect of H_2_ on activated Wnt/β-catenin signaling requires GSK3 activity. Then, we examined the effect of H_2_ in two cell lines, HCT-116 colon cancer cells and HepG2 liver carcinoma cells, which carry mutations in *CTNNB1* encoding β-catenin at its phosphorylation sites. In accordance with the guidelines by the Human Genome Organization (HUGO) (http://www.genenames.org/), *CTNNB1* and *Ctnnb1* are used in this communication to indicate the genes for β-catenin in human and mouse, respectively. HCT-116 cells are heterozygous for a deletion mutation at Ser45, the CK1 regulatory site, in the β-catenin gene^21^. HepG2 cells are heterozygous for an inframe-deletion lacking a potential GSK3-phosphorylation site in the β-catenin gene^22^. We found that the inhibitory effect of H_2_ on Wnt3a- or BIO-induced β-catenin accumulation in HCT-116 cells (Fig. 3c) was less than those observed in L cells (Fig. 1c,e) and HeLa cells (Supplementary Fig. S1e). The reduction of the H_2_ effect in HCT-116 cells was likely due to the presence of a deletion at Ser45. In HepG2 cells, Wnt3a or BIO failed to increase the truncated β-catenin and H_2_ showed no suppressive effect on the truncated β-catenin (Fig. 3d). In contrast, wild-type β-catenin in HepG2 cells was increased by Wnt3a or BIO, and was suppressed by H_2_ (Fig. 3d). To further confirm these results, we transfected L cells with HA-wild-type-β-catenin (HA-WT-β-catenin) or two β-catenin mutants (HA-ΔN-β-catenin and HA-S37A-β-catenin). HA-ΔN-β-catenin lacks the whole N-terminal region of β-catenin phosphorylated by CK1 and GSK3^23^. Another mutant HA-S37A-β-catenin has a Ser-to-Ala mutation at one of the GSK3-phosphorylated sites^23^. Enhanced Wnt/β-catenin signaling activity in L cells expressing HA-WT-β-catenin, but not HA-ΔN-β-catenin or HA-S37A-β-catenin, was decreased by H_2_ (Fig. 3e). These results indicate that CK1/GSK3-phosphorylation sites of β-catenin and the dual CK1/GSK3 activity are required for H_2_-medicated enhanced β-catenin degradation.

Next, we analyzed whether H_2_ regulates phosphorylation of glycogen synthase (GS), which is another substrate of GSK3. We found that H_2_ did not affect GSK3-mediated phosphorylation of GS in L cells (Supplementary Fig. S3a). Thus, H_2_ specifically enhances GSK3-mediated phosphorylation of β-catenin, but not GS.

In addition to kinase activities, phosphorylation status of β-catenin also depends on phosphatase activities. Okadaic acid, an inhibitor for protein phosphatase 2A (PP2A), promotes β-catenin hyperphosphorylation on serine-threonine residues^24^. To check whether PP2A mediates the effect of H_2_ on β-catenin degradation, L cells were pretreated with 30 nM okadaic acid and were added with BIO with 10% H_2_ or 10% N_2_ gas for 12 h. We observed that okadaic acid could not abrogate the suppressive effect of H_2_ on β-catenin accumulation in BIO-treated cells (Fig. 3f), indicating that H_2_ promoted β-catenin degradation independent of PP2A.

**Figure 4:**
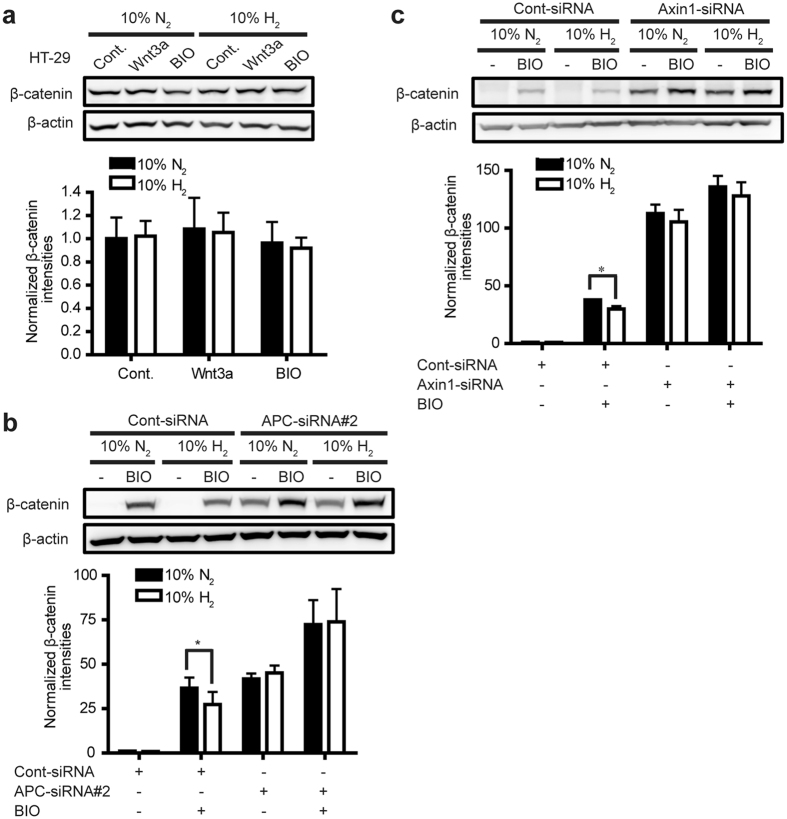
H_2_-mediated β-catenin degradation requires activities of APC and Axin1. **(a)** HT-29 cells carrying truncated APC were incubated with control CM (Cont.), Wnt3a CM, or 2 μM BIO with 10% H_2_ or 10% N_2_ gas for 24 h. Representative Western blots are shown with densitometry of β-catenin/β-actin (*n* = 3). No statistical difference by Student’s *t*-test. **(b)** L cells were transfected with Cont-siRNA or APC-siRNA#2, and treated with 2 μM BIO with 10% H_2_ or 10% N_2_ gas for 24 h. Representative Western blots are shown with densitometry of β-catenin/β-actin (*n* = 3). *P < 0.05 by Student’s *t*-test. **(c)** L cells were transfected with Cont-siRNA or Axin1-siRNA, and treated with 2 μM BIO with 10% H_2_ or 10% N_2_ gas for 24 h. Representative Western blots are shown with densitometry of β-catenin/β-actin (*n* = 3). *P < 0.05 by Student’s *t*-test.

In canonical Wnt/β-catenin signaling, two scaffold proteins, APC and Axin1, bind to β-catenin to form a degradation complex, and facilitate phosphorylation and ubiquitination of β-catenin. We then investigated whether APC, Axin1, or both are involved in H_2_-medicated β-catenin degradation. First, in HT-29 human colon cancer cells carrying truncated APC^21^, H_2_ failed to decrease β-catenin levels (Fig. 4a). Second, siRNA-mediated knock-down of APC in L cells abrogated the suppressive effect of H_2_ on β-catenin accumulation induced by BIO (Fig. 4b, Supplementary Fig. S3b,c). Third, similar to APC knock-down, knock-down of Axin1 also attenuated the effect of H_2_ (Fig. 4c and Supplementary Fig. S3d). In addition, H_2_ did not affect mRNA levels of APC and Axin1 (Supplementary Fig. S3e,f). Fourth, we conducted co-immunoprecipitation assay to examine the effect of H_2_ on the β-catenin degradation complex. To directly observe the effect of H_2_ on the degradation complex, we added MG132 to block β-catenin degradation in the proteasome pathway. We detected that H_2_ enhanced phosphorylation and ubiquitination of β-catenin in Axin1-immunoprecipitates, but had no effect on the interactions of Axin1 with β-catenin, APC, and GSK3 (Supplementary Fig. S3g,h). Taken together, H_2_ enhances phosphorylation of β-catenin mediated by CK1/GSK3 in the degradation complex formed by APC and Axin1, which subsequently leads to enhanced ubiquitination and degradation of β-catenin.

### H_2_ suppresses Wnt/β-catenin signaling in chondrocytes and protects against cartilage degradation in osteoarthritis (OA)

**Figure 5:**
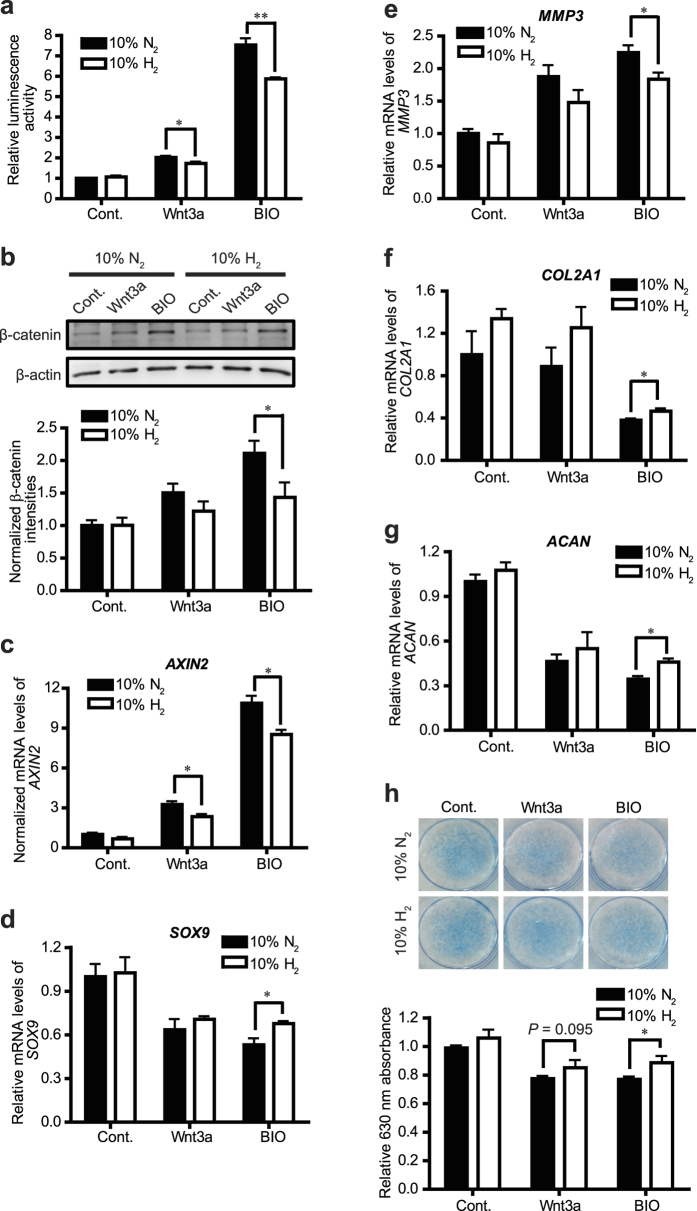
H_2_ inhibits Wnt/β-catenin signaling in chondrocytes. **(a)** HCS-2/6 human chondrosarcoma cells were treated with control CM (Cont.), Wnt3a CM, or 2 μM BIO with 10% H_2_ or 10% N_2_ gas for 24 h. The Wnt/β-catenin signaling activity was measured by Topflash luciferase reporter assay (*n* = 9). *P < 0.05 and **P < 0.01 by Student’s *t*-test. **(b)** Human OAC cells were treated with control CM (Cont.), Wnt3a CM, or 2 μM BIO with 10% H_2_ or 10% N_2_ gas for 24 h. Representative Western blots are shown with densitometry of β-catenin/β-actin (*n* = 4). *P < 0.05 by Student’s *t*-test. **(c–g)** Human OAC cells (clone 1) were treated with control CM (Cont.), Wnt3a CM, or 2 μM BIO with 10% H_2_ or 10% N_2_ gas for 24 h. Expression of *AXIN2* (*n* = 3) **(c)**, *SOX9* (*n* = 6) **(d)**, *MMP3* (*n* = 6) **(e)**, *COL2A1* (*n* = 3) **(f)**, and *ACAN* (*n* = 3) **(g)** were quantified by qRT-PCR. *P < 0.05 by Student’s *t*-test. **(h)** Differentiated ATDC5 cells were treated with control CM (Cont.), Wnt3a CM, or 2 μM BIO with 10% H_2_ or 10% N_2_ gas for 48 h. Proteoglycans were stained with Alcian blue (upper panel) and quantified by measuring the optical intensity at 630 nm of the cell lysates (*n* = 9 for Cont.-treated and BIO-treated cells, *n* = 6 for Wnt3a-treated cells). *P < 0.05 by Student’s t-test. Non-significant *P* values less than 0.10 are indicated above each pair.

Abnormal activation of Wnt/β-catenin signaling has been reported to be involved in the development and aggravation of OA^8^, which is characterized by degradation of ECM molecules, loss of articular cartilages, and formation of osteophytes. We then examined whether H_2_ inhibits Wnt/β-catenin signaling in HCS-2/8 human chondrosarcoma cells and human osteoarthritic chondrocyte (OAC) cells. We first confirmed that H_2_ downregulated Topflash reporter activity in HCS-2/8 cells stimulated by Wnt3a or BIO (Fig. 5a). Similarly, in human OAC cells stimulated by Wnt3a or BIO, H_2_ decreased β-catenin accumulation (Fig. 5b, Supplementary Fig. S4a,b) and suppressed mRNA levels of Axin2 (Fig. 5c, Supplementary Fig. S4a,b), but not of β-catenin (Supplementary Fig. S4c). Chondrocytes produce and maintain ECM, which mostly consists of collagens and proteoglycans. Dysfunction of articular chondrocytes in OA disturbs synthesis of ECM and enhances degradation of ECM. SRY-box 9 (Sox9), as a transcriptional factor, activates a number of cartilage ECM genes including *COL2A1* and *ACAN*^25^ and plays an essential role in chondrogenic differentiation. Physiological interaction between Sox9 and β-catenin results in their mutual degradation by the ubiquitin/proteasome system^26^. Increased level of β-catenin protein promotes degradation of Sox9 protein and vice versa^26^. We found that, in human OAC cells, stimulation of Wnt/β-catenin signaling by Wnt3a or BIO downregulated *SOX9* expression, which was partly rescued by H_2_ treatment (Fig. 5d, Supplementary Fig. S4a,b). H_2_ is thus expected to increase the level of Sox9 protein by suppressing Wnt/β-catenin signaling. This mechanism is likely to account for the H_2_-mediated up-regulation of Sox9 transcript, because Sox9 protein upregulates *SOX9* mRNA expression via forming a positive feedback loop^27^. We also compared gene expressions of other chondrogenic markers including *MMP3* encoding catabolic metalloproteinase 3, *COL2A1* encoding collagen type II α1, and *ACAN* encoding aggrecan in 3 clones of OAC cells derived from 3 patients (clone 1 in Fig. 5e–g, clone 2 in Supplementary Fig. S4a, and clone 3 in Supplementary Fig. S4b). In the absence of H_2_, we observed that Wnt3a and BIO induced expressions of the *MMP3*, *COL2A1*, and *ACAN* genes in variable directions from clone to clone. For example, BIO increased *MMP3* in clones 1 and 2, and decreased *MMP3* in clone 3. H_2_ changed expressions of these genes in favorable (downregulation of *MMP3*, and upregulation of *COL2A1* and *ACAN*) or unfavorable directions from clone to clone. To summarize, H_2_ suppresses Wnt/β-catenin signaling in human OAC cells, but the effect of H_2_ on ECM production cannot be evaluated due to variable responses of human OAC cells to Wtn3a and BIO. We also conducted Alcian blue staining in differentiated ATDC5 mouse chondrogenic cells, and found that both Wnt3a- and BIO-induced loss of proteoglycans were marginally reversed by H_2_ (Fig. 5h) without affecting proliferation of ATDC5 cells (Supplementary Fig. S4d).

**Figure 6:**
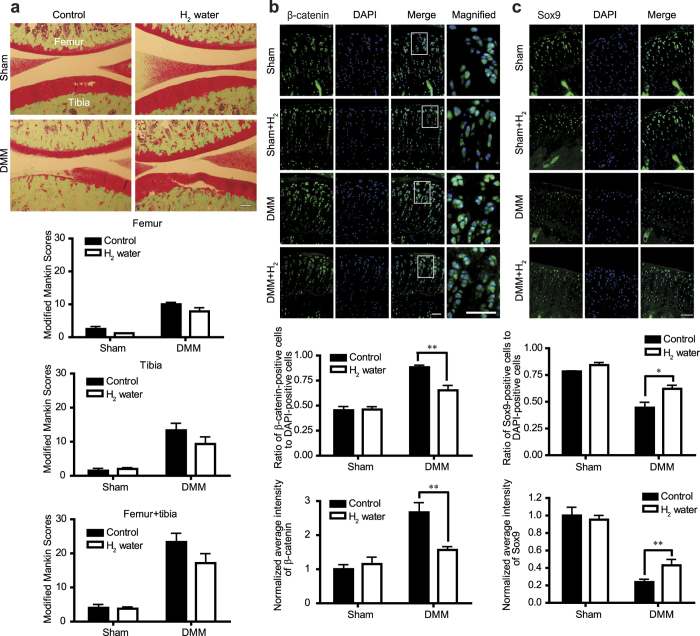
H_2_ modestly ameliorates cartilage degradation and improves expressions of β–catenin and Sox9 in a rat osteoarthritis (OA) model. **(a)** OA phenotype was generated by DMM surgery in Wistar/ST rats. Degassed water (control) or H_2_ water was administered *ad libitum* for 8 weeks after surgery. Each rat had DMM and sham surgeries in the right and left knees, respectively. Knee joints were stained with Safranin O and fast green, and representative images are shown (scale bar = 100 μm). Upper and lower sides of the image are femoral and tibial bones, respectively. Each image was blindly graded by modified Mankin score to evaluate severity of OA (sham groups, *n* = 5; DMM groups, *n* = 6). Mean and SEM are plotted. No statistical difference by Student’s *t*-test. **(b,c)** Immunofluorescence staining with antibodies against β-catenin **(b)** and Sox9 **(c)** in rat articular cartilage. Representative immunostaining images (scale bar = 50 μm) are shown with the ratio of β-catenin-positive cells (*n* = 9) **(b)** and Sox9-positive cells (*n* = 9) **(c)** to DAPI-positive cells, and average fluorescence intensity of β-catenin (*n* = 9) **(b)** and Sox9 (*n* = 9) **(c)**. Boxed regions in the merged images are magnified in the rightmost column to show nuclear translocation of β-catenin (scale bar = 50 μm) **(b)**. Three fields of the tibial cartilage area per section and 3 different sections per group were analyzed. Mean and SEM are plotted (*n* = 9). Intensity of β-catenin and Sox9 is normalized to the mean of the sham operated side of rats taking degassed water. *P < 0.05 and **P < 0.01by Student’s *t* test.

We next examined the effects of H_2_ on destabilization of the medial meniscus (DMM)-induced OA cartilage in rats, where Wnt/β-catenin signaling is abnormally activated^8^. Rats drank degassed water (control) or supersaturated H_2_ water (7 ppm) *ad libitum* from days 0 to 56 after DMM surgery. No difference in body weights was noted between the control and H_2_ groups (Supplementary Fig. S4e). We observed a tendency that 8-week administration of H_2_ water after surgery partially improved Safranin O-staining on the articular surface and minimally preserved the structure of articular cartilage (Fig. 6a). We also found that H_2_ decreased the percentage of β-catenin-positive cells and inhibited accumulation of β-catenin in cartilage chondrocytes in the DMM group without affecting β-catenin expression in sham groups (Fig. 6b). Additionally, we found that the expression of Sox9 was decreased in cartilage chondrocytes in the DMM group, which was partially rescued by H_2_ (Fig. 6c). These data indicate that H_2_ suppresses Wnt/β-catenin signaling in articular chondrocytes and partially ameliorates cartilage degradation and OA progression in a rat OA model.

## Discussion

Although more than 321 studies have demonstrated beneficial effects of H_2_ on both animal models and human diseases^1^, molecular target(s) of H_2_ have not been fully elucidated. H_2_ was initially reported as a selective scavenger of hydroxyl radical (•OH)^2^. However, H_2_ is a stable gas and the reaction rate constant of H_2_ and •OH is in the order of 10^7^ M^−1^·s^−1^, which is much lower than the reaction rate constants of •OH with other molecules (10^9^ to 10^10^ M^−1^·s^−1^)^28^. Additionally, the breath H_2_ concentration comes to the baseline level in 30 min after taking 200 ml saturated H_2_ water in healthy individuals^29^. As the reaction rate constant of H_2_ is low and the dwell time of H_2_ in our body is short, H_2_ is unlikely to efficiently remove •OH. Thus, yet unidentified mechanisms should underlie the therapeutic effect of H_2_. A previous report showed that oral intake of H_2_ water increases gastric secretion of ghrelin in mice^30^. In addition, intermittent, but not continuous, inhalation of H_2_ gas ameliorates a rat model of Parkinson’s disease^17^. We have also shown that H_2_ alters signaling activities in mast cells and macrophages without directly scavenging reactive oxygen/nitrogen species^3^,^4^.

In this study, we first showed that H_2_ inhibited endogenous β-catenin accumulation induced by Wnt3a and GSK3 inhibitors (LiCl and BIO), as well as exogenous β-catenin accumulation induced by a transgene, but had no effect on the basal endogenous β-catenin level. The effect of H_2_ was thus likely to be observed when Wnt/β-catenin signaling was abnormally activated. Then, we found that H_2_ increased β-catenin phosphorylation without attenuating the activity of PP2A, and accelerated β-catenin degradation without decreasing its mRNA level. Given that complete GSK3 inhibition and mutations at the CK1- or GSK3-phosphorylation sites of β-catenin nullified the H_2_ effect, the dual CK1 and GSK3 activities were required for H_2_-medicated β-catenin degradation. As H_2_ had no effect on GSK3-mediated phosphorylation of GS, which is another substrate of GSK3, H_2_ was likely to enhance GSK3 activity only in the degradation complex with APC and Axin1, but had no direct effect on GSK3 itself. Enhancement of CK1-mediated β-catenin phosphorylation by H_2_ is also in accordance with the assumption that the H_2_ works on the degradation complex, not on CK1 or GSK3. Additionally, as both CK1 and GSK3 are constitutively active in the resting state and as no allosteric activators are known, H_2_ is unlikely to be able to allosterically upregulate activities of CK1 and GSK3. To further confirm that the effect of H_2_ is on phosphorylation and not ubiquitination of β-catenin, we examined the effect of H_2_ on HT-29 human colon cancer cells harboring truncated APC. The truncated APC retains all three 15-aa repeats and three of seven 20-aa repeats, and lacks all three Axin1-binding sites and four of seven 20-aa repeats. A previous study shows that the three 20-aa repeats that are retained in the truncated APC are sufficient for β-catenin ubiquitination, and the interaction between the truncated APC and E3-ligase β-TrCP is not affected in HT-29 cells^21^. The Axin1-binding sites, which are deficient in HT-29 cells, markedly facilitate β-catenin phosphorylation, but are not required for β-catenin ubiquitination^21^,^31^. Thus, the low degradation rate of β-catenin in HT-29 cells is due to reduced phosphorylation of β-catenin because of lack of Axin1-binding sites^21^. Lack of the effect of H_2_ in HT-29 cells thus indicates that H_2_ enhances phosphorylation but not ubiquitination of β-catenin. In addition, our observation that knock-down of either APC or Axin1 minimizes the suppressive effect of H_2_ on β-catenin accumulation indicates that both APC and Axin1 are required for the H_2_ effect. We next examined the protein-protein interactions within the degradation complex using co-immunoprecipitation assay, and found that H_2_ had no effect on the interactions among Axin1, APC, β-catenin, and GSK3, but still could enhance CK1/GSK3-mediated phosphorylation of β-catenin. Water structure plays an active role in protein folding, enzyme catalysis, and cell signaling^32^. Alteration of the water structure will thus have a direct effect on biological systems. The H_2_O-H_2_ interaction accompanied by charge transfer is stronger than predicted^33^, which might affect the water structure. Additionally, as H_2_ can easily diffuse into every cellular compartment, solubilized H_2_ interfacing with other biomolecules possibly alters their hydration structures and subsequently their activities. In Wnt/β-catenin signaling, H_2_ may dynamically enhance protein-protein interactions during the process of β-catenin phosphorylation, which, however, could not be detected in our co-immunoprecipitation assays because of a rapid decrease of H_2_ concentration in a test tube^34^. Alternatively, H_2_ may modulate interaction of one of molecules constituting the degradation complex with a yet unidentified molecule, which leads to enhancement of β-catenin phosphorylation.

We have previously shown that H_2_ prevents degranulation of mast cells not by a radical scavenging effect, but by downregulating NADPH oxidase activity via attenuating the phosphorylation of the FcεRI-associated Lyn and its downstream signal transduction molecules (ERK1/2, JNK, p38 MAP kinase, and Akt) without affecting other signaling molecules [apoptosis signal-regulating kinase 1 (ASK1) and nuclear factor of kappa light polypeptide gene enhancer in B-cells inhibitor, alpha (IκB-α)]^3^. Similarly, in RAW264 macrophage cells, H_2_ reduces lipopolysaccharide/interferon γ (LPS/IFNγ)-induced nitric oxide (NO) release by suppressing the activity of inducible nitric oxide synthase (iNOS) not by affecting NADPH oxidase activity but by suppressing phosphorylation of ASK1 and its downstream signaling molecules (p38 MAP kinase, JNK, and IκB-α)^4^. We here demonstrate that H_2_ suppresses Wnt/β-catenin signaling without scavenging hydroxyl radicals or peroxynitrite. Inhibition of JNK, but not of ERK or p38 MAP kinase, suppresses Wnt3a-induced β-catenin accumulation^18^. However, the effect of H_2_ was unlikely to be dependent on JNK because inhibition of JNK did not attenuate the effect of H_2_. Akt phosphorylates GSK3 on Ser9 to inactivate it and inhibition of Akt activates GSK3^35^. Since we found that H_2_ had no effect on GSK3-mediated GS phosphorylation, it is unlikely that H_2_ activates GSK3 via suppression of Akt. Accordingly, H_2_ is able to specifically modulate signaling pathways in cell- and disease-specific manners.

In this study, we showed that H_2_ could not decrease abnormally elevated β-catenin in HCT-116, HepG2, and HT-29 cancer cells. Thus, H_2_ is unlikely to suppress proliferation of these cells. Previous studies have shown that H_2_ inhibited cell proliferation of human tongue carcinoma cells HSC-4^36^ and human fibrosarcoma cells HT-1080^36^. Similarly, a combination of H_2_ and 5-fluorouracil induced apoptosis of colon 26 cells^37^. Additionally, H_2_ suppressed the expression of vascular endothelial growth factor (VEGF) in human lung adenocarcinoma cells A549^38^. Although the effect of H_2_ on Wnt/β-catenin signaling has not been dissected in these cells, H_2_ might have achieved the tumor-suppressing effects by suppressing Wnt/β-catenin signaling. Alternatively, H_2_ might have suppressed cell proliferation by modulating other signaling pathway(s) and/or molecules.

As stated in introduction, abnormal activation of Wnt/β-catenin signaling deteriorates OA and thus Wnt/β-catenin signaling can be a therapeutic target for OA. In this study, we first show that H_2_ suppresses Wnt/β-catenin signaling in human OAC cells and also rescues Wnt3a- or BIO-induced loss of proteoglycan in differentiated ATDC5 chondrogenic cells. Additionally, H_2_ tended to protect against cartilage degradation in the DMM-induced OA model in rats, although statistical significance was not observed. The insignificant effect of H_2_ on the OA model may be partly accounted for by a limited effect of H_2_ on Wnt/β-catenin signaling in cartilage chondrocytes. Alternatively, the DMM-induced OA progression is accelerated by other signaling pathways that are insensitive to H_2_. In contrast to the deleterious effect of aberrantly activated Wnt/β-catenin signaling in OA, excessive inhibition of Wnt/β-catenin signaling also worsens OA^39^, possibly due to an essential role of Wnt/β signaling in cartilage development and homeostasis^40^. As H_2_ did not change the levels of endogenous β-catenin and Sox9 on the sham-operated side, H_2_ is expected to have no effect on cartilage development and homeostasis. Our results suggest that H_2_ may be able to attenuate OA progression in humans. However, other signaling pathways and/or molecules, including transforming growth factor β (TGF-β) signaling and inflammatory responses, are also involved in OA development and progression^41^. In this study, we could not exclude the possibility that H_2_ modulated other signaling pathways or molecules to ameliorate OA.

**Figure 7:**
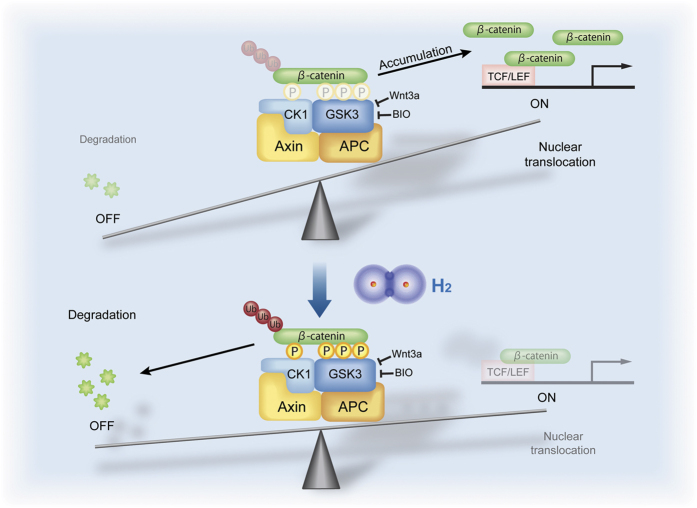
Schematic diagram showing that H_2_ promotes phosphorylation and degradation of β-catenin in activated Wnt/β-catenin signaling. β-catenin is phosphorylated at one site by CK1 and at three sites by GSK3 in the degradation complex composed of CK1, GSK3, APC, and Axin1. Wnt3a or BIO inhibits phosphorylation and ubiquitination of β-catenin, and increases the intracellular level of β-catenin, which drives Wnt/β-catenin signaling from the “off-state” to the “on-state”. H_2_ works on the degradation complex to enhance phosphorylation and degradation of β-catenin when Wnt/β-catenin signaling is activated by Wnt3a or BIO, and moves the “on-state” toward the “off-state”. H_2_ has no direct effect on the protein phosphatase 2A (not shown) or the ubiquitination process.

We present that H_2_ suppresses abnormally activated Wnt/β-catenin signaling, which plays pivotal roles in diverse pathophysiologic processes, by enhancing β-catenin phosphorylation in the degradation complex (Fig. 7). For a considerable fraction of the 166 disease models and human diseases, for which the effects of H_2_ have been documented^1^, H_2_ is likely to have exerted beneficial effects by suppressing Wnt/β-catenin signaling.

## Materials and Methods

### Cell culture with H_2_ gas

Cells were cultured in a culture dish in a 560-ml closed plastic box that was covered with aluminum and humidified with water at the base of the box. The box was put in a convection incubator (SLI-221, EYELA). We adjusted the temperature of the incubator to make the temperature inside the box 37 °C using an electronic thermometer. In the H_2_ group, H_2_ gas (3 ml/min or 6 ml/min) was mixed with CO_2_-added air (5% CO_2_ and 95% air, 60 ml/min) to make 5% or 10% H_2_ gas, which was delivered into the box via an afferent tube and out of the box via an efferent tube connected to a draft chamber. In the control group, 3 ml/min or 6 ml/min of N_2_ gas was used instead of H_2_ gas to control for O_2_ concentrations. We measured the hydrogen concentration in the medium by equilibrating 1 ml medium with 100 ml of 100% N_2_ gas and by injecting 1 ml equilibrated gas into a gas chromatography connected to a semiconductor gas detector (EAGanalyzer GS-23, SensorTec). As shown in Supplemental Fig. S1i, H_2_ concentrations were detectable in culture medium in 2 min after administration of H_2_ gas. The H_2_ concentration in the culture medium stayed stable after 20 min of H_2_ administration. The amount of H_2_ dissolved in the culture medium doubled by increasing H_2_ concentration from 5% to 10%. The pH of the culture medium after culturing L cells for 24 h with 10% N_2_ or 10% H_2_ gas administration was 7.71 ± 0.03 and 7.67 ± 0.02 (mean and SEM, *n* = 3), respectively, with no statistical difference by Student’s *t*-test.

### Cell culture, chemicals, and reporter assay

L, L Wnt3a, HeLa, HCT-116, and HT-29 cells were obtained from ATCC. HepG2 and ATDC5 cells were from RIKEN BioResource Center. HCS-2/8 cells were kindly provided by Dr. Masaharu Takigawa at Okayama University. Studies using human OAC cells were approved by the Ethical Review Committee of Nagoya University Graduate School of Medicine, and were performed in accordance with the relevant guidelines by MHLW, Japan. After a written informed consent was given, OAC cells were obtained from patients who underwent total joint replacement for severe knee OA. L, L Wnt3a, HeLa, HCT-116, HT-29, HepG2, HCS-2/6, and human OAC cells were cultured in the Dulbecco’s Modified Eagle’s medium (DMEM, Gibco) supplemented with 10% fetal bovine serum (FBS, Thermo Scientific). ATDC5 cells were cultured in DMEM/F12 (a mixture of Dulbecco’s modified Eagle’s medium and Ham’s F12 medium, Sigma–Aldrich) supplemented with 5% FBS. L and L Wnt3a cells were cultured for 4 d to make control conditioned medium (CM) and Wnt3a CM, respectively. Lithium chloride (LiCl), cycloheximide (CHX), N-benzyoloxycarbonyl (Z)-Leu-Leu-leucinal (MG132), dimethyl sulfoxide (DMSO), and SP600125 were purchased from Wako. Okadaic acid was bought from Research Biochemicals International (RBI), and 6-bromoindirubin-3′-oxime (BIO) was from Sigma-Aldrich. To quantify the canonical Wnt/β-catenin signaling activity, cells were transfected with the Topflash luciferase reporter plasmid (M50 Super 8 × Topflash plasmid, Addgene) and the *Renilla* luciferase plasmid (phRL-TK, Promega). L cells were transfected using Lipofectamine 2000 (Invitrogen), whereas HeLa and HCS-2/8 cells were transfected using FuGENE 6 (Roche). Twenty-four hours later, cells were treated with either 50% control CM, 50% Wnt3a CM, 30 mM LiCl, or 2–4 μM BIO in 10% H_2_ or 10% N_2_ gas for 24 h. Luciferase activity was measured in triplicate by the Dual Luciferase Reporter Assay System (Promega).

### Plasmids, siRNAs, and transfection

A plasmid carrying myc-β-catenin (XE28 XBC 40) was kindly gifted from Dr. Takamasa Yamamoto at National Institute for Basic Biology. Plasmid carrying HA-WT-β -catenin, HA-ΔN-β-catenin, and HA-S37A-β-catenin were kindly provided by Dr. Eisuke Nishida at Kyoto University. L cells were transfected with plasmids using Lipofectamine 2000 (Invitrogen). Sequences of siRNAs against APC and Axin1 were adopted from previous studies^42^,^43^ and were synthesized by Sigma-Aldrich. To knock-down APC, L cells were transfected with 280 pmol APC-targeting siRNA (APC-siRNA#1 or APC-siRNA#2) or control siRNA (Cont-siRNA) by Lipofectamine RNAiMax (Invitrogen). Forty-eight hours later, cells were treated with 2 μM BIO with 10% H_2_ or 10% N_2_ gas for 24 h. To knock-down Axin1, we performed siRNA transfection in two consecutive days as described previously^43^. Briefly, L cells were transfected with 140 pmol Axin1-targeting siRNA (Axin1-siRNA) or Cont-siRNA by Lipofectamine RNAiMax, and on the second day we repeated the same steps. Twenty-four hours later, cells were treated with 2 μM BIO with 10% H_2_ or 10% N_2_ gas for 24 h.

### Preparation of cell lysates and Western blotting

Cells were washed with PBS twice and were harvested with PLC buffer containing 50 mM HEPES (pH 7.0), 150 mM NaCl, 10% glycerol, 1% TritonX-100, 1.5 mM MgCl_2_, 1 mM EGTA, 100 mM NaF, 10 mM sodium pyrophosphate, 1 μg/μl aprotinin, 1 μg/μl leupeptin, 1 μg/μl pepstatin A, 1 mM PMSF, 1 mM sodium orthovanadate, and the Phosphatase Inhibitor Cocktail (PhosSTOP, Roche). The lysates were incubated on ice for 15 min, sonicated for 10 sec, and then centrifuged at 20,600 × *g* at 4 °C for 15 min. Preparation of nuclear fraction is described in details in the Supplementary information. The total protein concentrations of the lysates were measured by Pierce 660 nm Protein Assay Reagent. Cell lysates were boiled for 5 min in 2 × Laemmli buffer, separated on a 7.5% or 10% SDS-polyacrylamide gel, and transferred to a polyvinylidene fluoride membrane (Immobilon-P, Millipore). Membranes were washed in Tris-buffered saline containing 0.05% Tween 20 (TBS-T) and blocked for 1 h at room temperature in TBS-T with 5% skim milk or 5% bovine serum albumin (BSA). The membranes were then incubated overnight at 4 °C with specific antibodies listed in Supplementary Table S1. The membranes were washed with TBS-T and incubated with secondary goat anti-mouse IgG (1: 5000, LNA931V/AG, GE Healthcare) or anti-rabbit IgG (1: 5000, LNA934V/AE, GE Healthcare) antibody conjugated to horseradish peroxidase (HRP) for 1 h at room temperature. The bound antibodies were detected with Amersham ECL Western blotting detection reagents (GE Healthcare), and the signal intensities were quantified with the ImageJ program.

### *In vivo* ubiquitination assay

Proteins were immunoprecipitated from L cell lysates as shown in the Supplementary information.

### Gene expression analysis

Total RNAs from cells were extracted by RNeasy Mini Kit (Qiagen) according to the manufacturer’s instructions and then reverse-transcribed into complementary DNA (cDNA) using an oligo-dT primer (Thermo Fisher Scientific) and ReverTraAce (Toyobo). Real-time quantitative PCR (qPCR) was performed with the LightCycler 480 (Roche Applied Science) using the SYBR Premix ExTaq (Takara Bio). The expression level of a specific gene was normalized by the levels of β2-microglobulin (β2-MG). PCR primers are shown in Supplementary Table S2. All real-time qPCR experiments were performed in triplicate.

### Co-immunoprecipitation

Details are shown in the Supplementary information.

### Alcian blue staining

Alcian blue staining in differentiated ATDC5 cells was performed as described previously with slight modifications^8^. Details are shown in the Supplementary information.

### Cell proliferation assay

Cell proliferation of ATDC5 cells were estimated by a BrdU cell proliferation ELISA kit (Roche). Details are shown in the Supplementary information.

### Animal experiments and administration of supersaturated hydrogen water

All animal studies were approved by the Animal Care and Use Committee of the Nagoya University Graduate School of Medicine, and were performed in accordance with the relevant guidelines by MEXT, Japan. Details of experiments with fed and starved mice are shown in the Supplemental information. For generation of osteoarthritis (OA) model, eight-week-old male Sprague-Dawley rats were purchased from Japan SLC. Rats were anesthetized with isopropentane. The OA model was generated by resection of the menisco-tibial ligament to destabilize medial meniscus (DMM) in the right knee. On the sham-operated left side, the skin and joint capsule was incised and sutured. Rats were randomly divided into 2 groups: the degassed water group (control) and the H_2_ water group with unlimited access to degassed water and H_2_ water after surgery, respectively. H_2_ water was freshly prepared every evening using Hydrogen Water 7.0 (Ecomo International), which was kindly provided by MiZ Co. Ltd. The concentration of dissolved H_2_ was 5 to 7 ppm, whereas the concentration of saturated H_2_ under the standard ambient temperature and pressure (SATP) is 1.6 ppm. The H_2_ concentrations in the glass vessel for rodents exponentially decreased with a half-life of 1.09 h^34^. As rodents drink water every hour at night, the average H_2_ concentration in the water that the rodents drank was predicted to be 1.66 ppm^34^. Rats were sacrificed 8 weeks after surgery. Tissues around the knees were fixed overnight in 4% paraformaldehyde at 4 °C, dehydrated, and embedded in paraffin. Safranin O and fast green stainings were performed on the sagittal sections. The modified Mankin histologic scores on both tibial and femoral sides of articular cartilages to estimate severity of OA were graded by 2 blinded investigators as described previously^44^. One well-cut and well-stained sagittal section in the medial region of the joint per joint was scored. Details of immunofluorescence staining of β-catenin and Sox9 are shown in the Supplementary information.

### Statistical analysis

All values were presented as the mean and SEM. For *in cellulo* studies, values are normalized to those of cells treated with 50% control CM and 10% N_2_ gas, unless otherwise indicated. Statistical significance was estimated either by Student’s *t*-test or two-way repeated measures ANOVA test. Bonferroni correction was applied to Student’s *t*-test for multiple comparisons. *P*-values less than 0.05 were considered significant.

## Additional Information

**How to cite this article**: Lin, Y. *et al*. Molecular hydrogen suppresses activated Wnt/β-catenin signaling. *Sci. Rep.* **6**, 31986; doi: 10.1038/srep31986 (2016).

## Supplementary Material

Supplementary Information
