# Supplementary material for: Molecular hydrogen suppresses activated Wnt/β-catenin signaling
Source: Sci Rep. 2016 Aug 25;6:31986. doi: 10.1038/srep31986 (PMC5001535; doi:10.1038/srep31986)
Supplement: Supplementary Information [file srep31986-s1.pdf]

Molecular hydrogen suppresses activated Wnt/β-catenin signaling

Yingni Lin[1], Bisei Ohkawara[1], Mikako Ito[1], Nobuaki Misawa[2], Kentaro Miyamoto[1], Yasuhiko Takegami[1], Akio Masuda[1], Shinya Toyokuni[2], Kinji Ohno[1,*]

[1]Division of Neurogenetics, Center for Neurological Diseases and Cancer, Nagoya University Graduate School of Medicine, Nagoya, Japan

[2]Department of Pathology and Biological Responses, Graduate school of Medicine, Nagoya University Graduate School of Medicine, Nagoya, Japan

*Address correspondence to Dr. Kinji Ohno

Division of Neurogenetics, Center for Neurological Diseases and Cancer, Nagoya University Graduate School of Medicine, 65 Tsurumai, Showa-ku, Nagoya 466-8550, Japan.

e-mail: ohnok@med.nagoya-u.ac.jp

Supplementary information includes:

Supplementary materials and methods.

Supplementary Tables S1-S2.

Supplementary Figures S1-S4.

Supplementary material and methods

Preparation of nuclear fraction

Cells were trypsinized, washed with PBS and resuspended in buffer A containing 10 mM HEPES-KOH (pH 7.8), 10 mM KCl, 0.1 mM EDTA and 0.1% NP-40, 10 mM sodium pyrophosphate, 1 μg/μl aprotinin, 1 μg/μl leupeptin, 1 μg/μl pepstatin A, 1 mM PMSF, 1 mM sodium orthovanadate, and the Phosphatase Inhibitor Cocktail. The cells were then vortexed and centrifuged at 5000 x *g* for 2 min. Supernatant was removed, and the pellet was washed with buffer A and resuspended in buffer C containing 50 mM HEPES-KOH (pH 7.8), 420 mM KCl, 0.1 mM EDTA, 5 mM $MgCl_2$, 2% glycerol, 10 mM sodium pyrophosphate, 1 μg/μl aprotinin, 1 μg/μl leupeptin, 1 μg/μl pepstatin A, 1 mM PMSF, 1 mM sodium orthovanadate, and the Phosphatase Inhibitor Cocktail. The pellet was vortexed and incubated at 4 °C on a rotating shaker for 30 min. The pellet was then centrifuged at at 20,600 x *g* at 4 ºC for 15 min. The supernatant was collected as nuclear fraction.

In vivo experiments with fed and starved mice

Eight-week-old C57BL/6J mice were purchased from Japan SLC. Mice were randomly divided into 4 groups: free access to degassed water and food before euthanasia (fed control group), free access to hydrogen water and food before euthanasia (fed+H_2 group), free access to degassed water and starved before euthanasia (starved control group), and free access to hydrogen water and starved before euthanasia (starved+H_2 group). Mice had free access to either degassed water or H_2 water for 10 d. Mice had free access to food up to euthanasia or were starved for 18 h before euthanasia. The liver was isolated, flash-frozen in liquid nitrogen, and stored at -80 °C until further applications. For protein extraction, liver tissues were homogenized and lysed in RIPA buffer (Thermo Scientific Pierce, 89900) followed by centrifugation for 15 min at 20,600 x *g* at 4 °C.

In vivo ubiquitination assay

Cells were harvested by a lysis buffer containing 2% SDS, 150 mM NaCl, 10 mM Tris-HCl (pH 8.0) with 2 mM sodium orthovanadate, 1 mM PMSF, 50 mM NaF, 1 μg/μl pepstatin A, 1mM DTT and the Protease Inhibitor Cocktail, and boiled for 10 min. Samples were then diluted in a dilution buffer (1: 10) containing 10 mM Tris-HCl (pH 8.0), 150 mM NaCl, 2 mM EDTA, 1% TritonX-100, incubated at 4 °C for 30 min on a rotating shaker, and centrifuged at 20,000 x *g* for 30 min. The total protein concentrations of the supernatants were measured by Pierce

660 nm Protein Assay Reagent, and 500 μg protein were incubated with anti-β-catenin antibody at 4 °C on a rotating shaker overnight, followed by addition of Dynabeads Protein G (Invitrogen) for another 1 h. Protein-antibody-bound beads were washed with a washing buffer [10 mM Tris-HCl (pH 8.0), 150 M NaCl, 1 mM EDTA, 1% NP-40] and boiled for 5 min with standard 2× Laemmli buffer. The immunoprecipitates were separated by SDS-PAGE and immunoblotted with anti-ubiquitin and anti-β-catenin antibody, as stated in Supplementary Table S1.

Co-immunoprecipitation

Cells were lysed in a buffer containing 20 mM Tris pH 8.0, 140 mM NaCl, 1 mM EDTA, 10% glycerol, 1% Triton X-100, 1mM DTT, 50 mM NaF, 1 mM PMSF, 1 mM sodium orthovanadate, 1 μg/μl pepstatin A, and the Phosphatase Inhibitor Cocktail. After centrifugation for 15 min at 20,600 x *g* at 4 °C, supernatants were collected and protein concentrations were measured by Pierce 660 nm Protein Assay Reagent. Lysates with the same amounts of proteins (750-1000 μg) were incubated with 1.0-1.5 μg of an antibody against Axin1 (05-1579, Millipore) or control mouse IgG (sc-2025, Santa Cruz Biotechnology) overnight at 4 °C. Dynabeads protein G (Invitrogen) were blocked with 1 μg/ml BSA for 1 h at 4 °C, added to the lysates with indicated antibody, and incubated for 1 h. Protein-antibody-bound beads were washed with PBS for 4 times and resuspended in standard 2× Laemmli buffer. Immunoprecipitated proteins were analyzed by Western blotting using specific antibodies against phospho-β-catenin (Ser45), phospho-β-catenin (Ser33/Ser37/Thr41), β-catenin, APC, GSK-3α/β, and Axin1, as stated in Supplementary Table S1.

Alcian blue staining

ATDC5 cells were seeded in a 12-well plate, and were differentiated into chondrocytes with insulin-transferrin-selenite (ITS, Invitrogen) for 21 days. Cells were treated with either 50% control CM, 50% Wnt3a CM, or 2 μM BIO with 10% H_2 or 10% N_2 gas for 48 h. Then, cells were fixed with methanol for 30 min at -20 °C, and stained overnight with 0.5% Alcian Blue 8 GX (Sigma) in 1N HCl. The stained cells were lysed in 200 μl or 400 μl of 6 M guanidine HCl for 6 h at room temperature. The optical density of the extracted Alcian blue was measured at 630 nm using PowerScan 4 (DS Pharma Biomedical).

Cell proliferation assay

Proliferation of ATDC5 cells was estimated by the BrdU cell proliferation ELISA kit (Roche) according to the manufacturer's recommendations. Briefly, ATDC5 cells were seeded at 5×10^3/well in a 96-well plate and were incubated with 10% H_2 or 10% N_2 gas for 48 h. Then, cells were labeled with 10 μM BrdU for 2 h at 37 °C, fixed and incubated with anti-BrdU antibody for 90 min at room temperature. After incubating the lysate with the substrate solution for 10 min, the reaction was stopped by H_2SO_4. Absorbance was measured at 450 nm using an absorbance microplate reader (Sunrise Remote, Tecan).

Immunofluorescence staining

For immunofluorescence staining, the paraffin-embedded sections were first deparaffinized and rehydrated. Then, the sections were unmasked by 10 mM sodium citrate buffer (pH 6.0) at 95 °C for 15 min and were incubated with 3% H_2O_2 for 15 min to inactivate endogenous peroxidases. Sections were blocked with 5% goat serum in TBS-T for 1 h at room temperature followed by overnight incubation with antibodies against β-catenin (1: 100 in 5% goat serum with TBS-T, #9587, Cell Signaling Technology) or Sox9 (1: 400 in 5% goat serum with TBS-T, AB5535, Chemicon International) at 4 °C. After washing with TBS-T, sections were incubated with Alexa Fluor 488 donkey anti-rabbit IgG (1: 500, Invitrogen) for 1 h at room temperature. Finally, the specimens were mounted in VectaShield containing 2 μg/ml diamidino-2-phenylindole (DAPI) (Vector Laboratories). The percentage of β-catenin-positive cells and Sox9-positive cells to DAPI-positive cells, and average signal intensities of β-catenin and Sox9 were blindly estimated using MetaMorph (Molecular Device). Three fields of the tibial cartilage area per section and 3 different sections per group were analyzed.

Table S1. Antibodies and concentrations for Western blotting

Name	Manufacturer	Clone	Cat. No.	Dilution
β-catenin	BD Transduction Laboratories	Mouse monoclonal Clone 14	610153	1: 2000
c-myc	Santa Cruz Biotechnology	Mouse monoclonal Clone 9E10	sc-40	1: 500
ubiquitin	Santa Cruz Biotechnology	Mouse monoclonal Clone P4D1	sc-8017	1: 400
β-actin	Santa Cruz Biotechnology	Mouse monoclonal Clone C-4	sc-47778	1: 400
GSK-3α/β	Santa Cruz Biotechnology	Mouse monoclonal Clone 1H8	sc-7291	1:200
Axin1	Millipore	Mouse monoclonal Clone A5	05-1579	1: 1000
phospho-c-Jun (Ser73)	Cell Signaling Technology	Rabbit monoclonal Clone D47G9	#3270	1: 1000
c-Jun	Santa Cruz Biotechnology	Rabbit polyclonal Clone H-79	sc-1694	1:200
phospho-SAPK/JNK (p-JNK) (Thr183/Tyr185)	Cell Signaling Technology	Rabbit monoclonal Clone 81E11	#4668	1: 1000
SAPK/JNK (JNK)	Cell Signaling Technology	Rabbit polyclonal	#9252	1: 1000
phospho-β-catenin (Ser45)	Cell Signaling Technology	Rabbit polyclonal	#9564	1: 1000
phospho-β-catenin (Ser33/Ser37/Thr41)	Cell Signaling Technology	Rabbit polyclonal	#9561	1: 1000
glycogen synthase (GS)	Cell Signaling Technology	Rabbit polyclonal	#3893	1: 1000
phospho-GS (Ser641)	Cell Signaling Technology	Rabbit polyclonal	#3891	1: 1000
lamin B1	Abcam	Rabbit polyclonal	ab16048	1: 1000
APC	Santa Cruz Biotechnology	Rabbit polyclonal Clone C-20	sc-896	1: 200

Table S2. Primer sequences for qRT-PCR

Gene	Forward primer (5'-3')	Reverse primer (5'-3')
Mouse Axin2	GAAACTGGCAAGTGTCCACG	CGCAGGCAAATTCGTCACTC
Human *AXIN2*	CTCCCCACCTTGAATGAAGA	GTTTCCGTGGACCTCACACT
Mouse *Axin1*	GGAGCTATTCCGAGAACGCA	ACTGGGTACCTCAGCATTGG
Mouse *Apc*	TCCCTTCGCTCCTACGGAA	TGAGCATAATACCAGTCCTTTTCCT
Mouse *Ctnnb1*	TTAAACTCCTGCACCCACCAT	AGGGCAAGGTTTCGAATCAA
Human *CTNNB1*	GCTTTCAGTTGAGCTGACCA	CAAGTCCAAGATCAGCAGTCTC
Human *MMP3*	GCAGTTTGCTCAGCCTATCC	GAGTGTCGGAGTCCAGCTTC
Human *SOX9*	CTGGGCAAGCTCTGGAGA	ATGTGCGTCTGCTCCGTG
Human *ACAN*	CCAGGAGGTATGTGAGGA	CGATCCACTGGTAGTCTTG
Human *COL2A1*	AGAGGGGATCGTGGTGACAA	GGCAGCAAAGTTTCCACCAA
Mouse *B2m*	ATTCACCCCCACTGAGACTG	TGCTATTTCTTTCTGCGTGC
Human *B2M*	TGGCACCTGCTGAGATACTG	AGGATGCTAGGACAGCAGGA

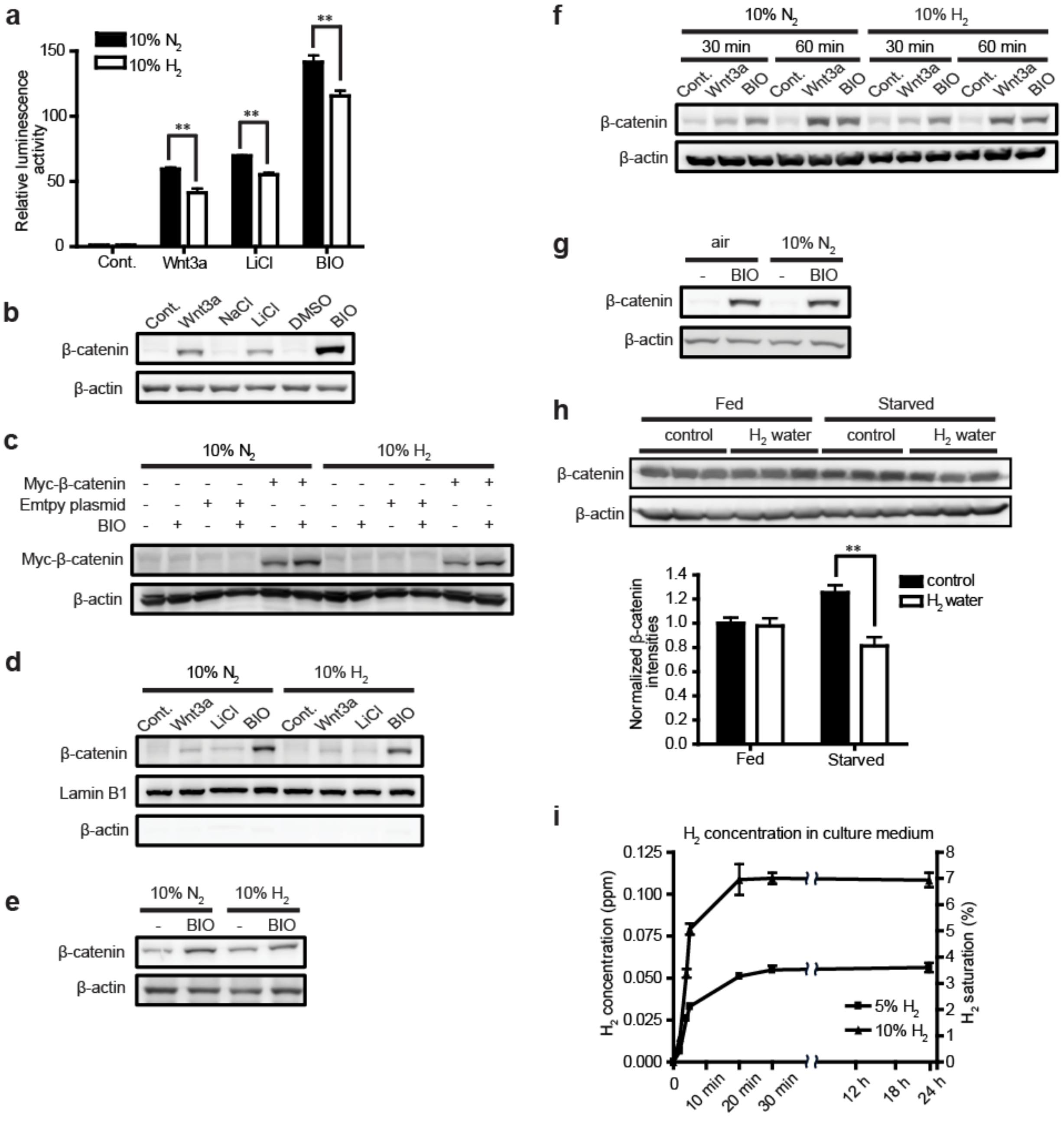

Supplemental Figure 1. H_2 suppresses Wnt/β-catenin signaling.

(a) HeLa cells were treated with control CM (Cont.), Wnt3a CM, 30 mM LiCl, or 4 μM BIO with 10% H_2 or 10% N_2 gas for 24 h. The Wnt/β-catenin signaling activity was measured by Topflash luciferase reporter assay (n = 3). Mean and SEM are plotted. Values are normalized to the mean of Cont. and 10% N_2. $^{**}P <$ 0.01 by Student's t test.

(b) L cells were treated with pairs of control CM (Cont.) and Wnt3a CM; 30 mM NaCl and 30 mM LiCl; and 0.02% DMSO and 2 μM BIO/0.02% DMSO for 24 h. Representative Western blots are shown.

(c) L cells were transfected with an empty or myc-β-catenin plasmid. Cells were treated with 2 μM BIO with

10% H_2 or 10% N_2 gas for 24 h. Representative Western blots with anti-c-myc antibody are shown.

(d) L cells were treated with control CM (Cont.), Wnt3a CM, 30 mM LiCl, or 2 µM BIO with 10% H_2 or 10% N_2 gas for 24 h. Representative Western blots with nuclear fractions are shown.

(e) HeLa cells were treated with 4 µM BIO with 10% H_2 or 10% N_2 gas for 24 h. Representative Western blots with anti-β-catenin antibody are shown.

(f) L cells were treated with control CM (Cont.), Wnt3a CM, or 2 µM BIO with 10% H_2 or 10% N_2 gas for 30 min and 60 min. Representative Western blots with anti-β-catenin antibody are shown.

(g) L cells were treated with 2 µM BIO with air or 10% N_2 gas for 24 h. Representative Western blots with anti-β-catenin antibody are shown.

(h) C57BL/6J mice were given degassed water (control) or H_2 water *ad libitum* for 10 d and were fed or starved for 18 h before sacrifice. Representative Western blots of the liver of 3 mice in each group are shown with densitometry of β-catenin/β-actin (n = 6 in each group). Values are normalized to control fed group. $^{**}P < 0.01$ by Student's t test.

(i) Time course analysis of H_2 concentrations in culture medium treated with 5% or 10% H_2 gas for indicated time periods (n = 3). Mean and SEM are plotted. Note that the concentration of saturated H_2 under standard ambient temperature and pressure (SATP) is 1.6 ppm.

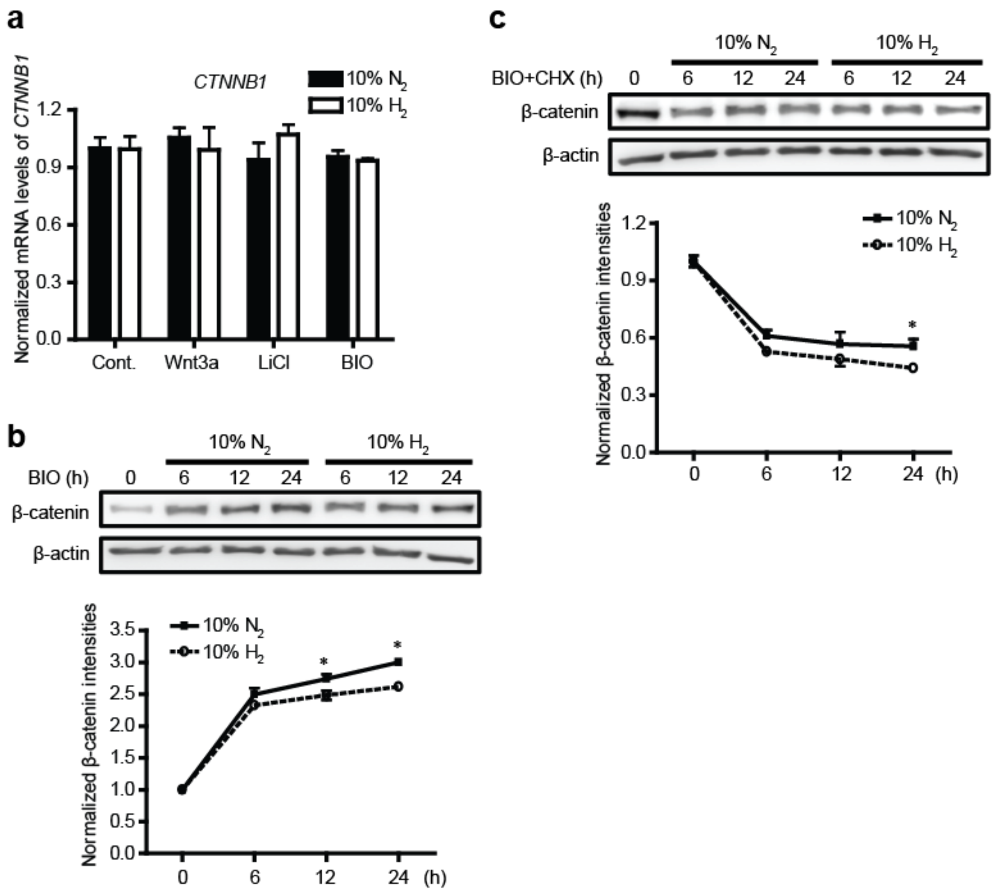

Supplemental Figure 2. H_2 promotes degradation of endogenous β-catenin without decreasing its mRNA level in HeLa cells.

(a) HeLa cells were treated with control CM (Cont.), Wnt3a CM, 30 mM LiCl, or 4 μM BIO with 10% H_2 or 10% N_2 gas for 24 h. Expression of *CTNNB1* encoding β-catenin was quantified by qRT-PCR ($n = 3$). No statistical difference by Student's *t*-test.

(b, c) HeLa cells were added with 4 μM BIO **(b)** or a combination of 10 μg/ml CHX and 4 μM BIO **(c)** with 10% H_2 or 10% N_2 gas for indicated periods of time. Representative Western blots are shown with densitometry of β-catenin/β-actin ($n = 3$). Two groups in each panel were not statistically different by two-way repeated measures ANOVA [$P = 0.071$ for **(b)** and $P = 0.0531$ for **(c)**]. *$P < 0.05$ by Student's *t*-test with Bonferroni correction for each pair of H_2 and N_2. Values are normalized to the mean of 10% N_2 at 0 h.

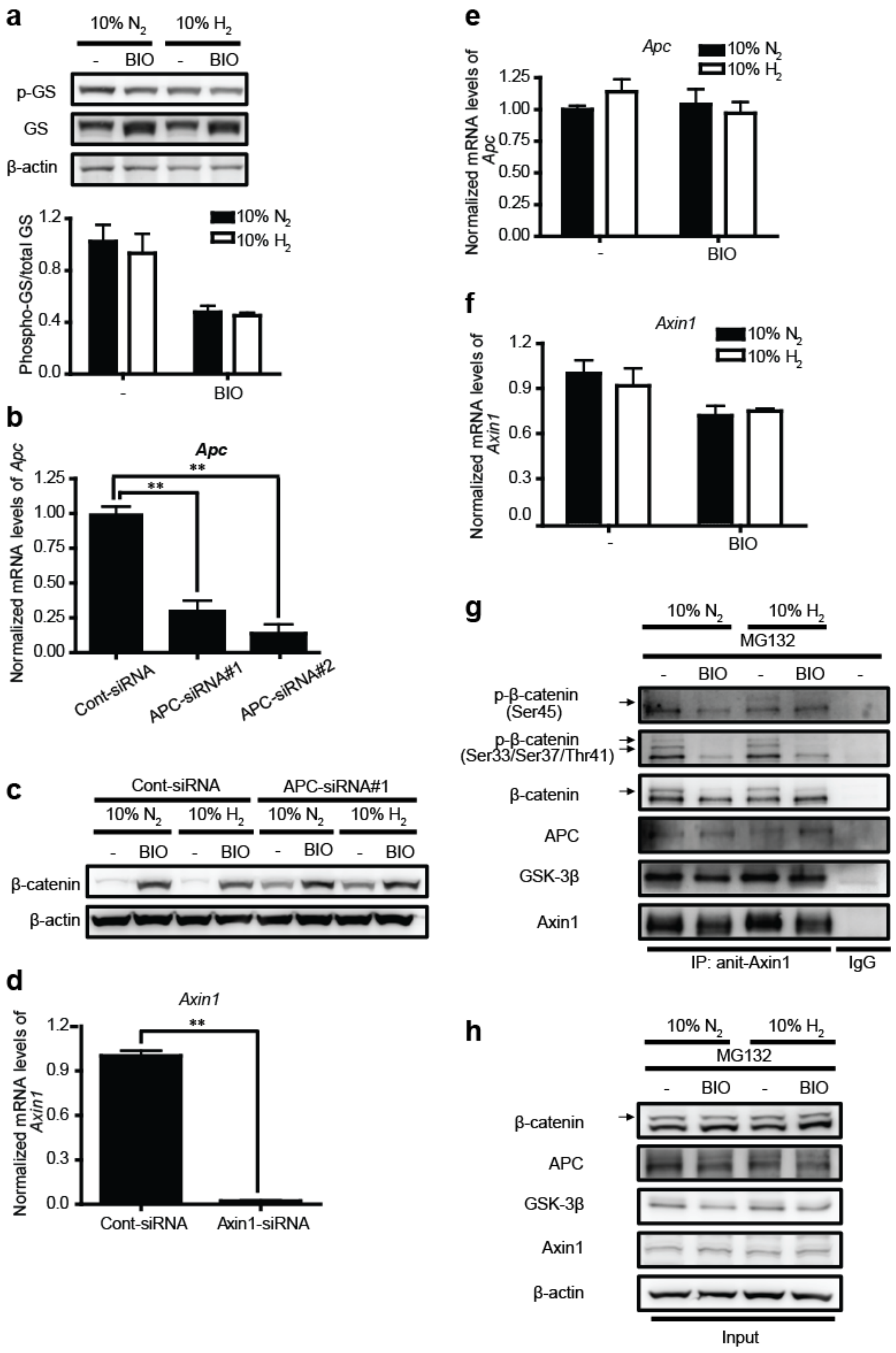

Supplemental Figure 3. H_2-mediated β-catenin degradation requires activities of APC and Axin1.

(a) L cells were treated with 2 µM BIO with 10% H_2 or 10% N_2 gas for 24 h. Western blots are shown with densitometry of phospho-glycogen synthase (p-GS)/glycogen synthase (GS) (n = 3). No statistical difference by Student's t-test.

(b) Knock-down efficiencies of endogenous APC by two siRNAs were assessed by qRT-PCR in L cells (n = 3).

$^{**}P < 0.01$ by Student's *t*-test.

(c) L cells were transfected with Cont-siRNA or APC-siRNA#1, and were treated with 2 μM BIO with 10% H_2 or 10% N_2 gas for 24 h. Representative Western blots are shown.

(d) Knock-down efficiency of endogenous Axin1 by siRNA was assessed by qRT-PCR in L cells ($n = 3$). $^{**}P < 0.01$ by Student's *t*-test.

(e, f) L cells were treated with 2 μM BIO with 10% H_2 or 10% N_2 gas for 24 h. Expressions of *Apc* ($n = 3$) **(e)** and *Axin1* ($n = 3$) **(f)** were quantified by qRT-PCR. No statistical difference by Student's *t*-test.

(g, h) L cells were treated with 10 μM MG132 in the presence or absence of 2 μM BIO with 10% H_2 or 10% N_2 gas for 12 h. Cell lysates were then immunoprecipitated (IP) with antibodies against Axin1 or mouse control IgG (IgG). Representative Western blots of IP samples **(g)** and input samples **(h)** with indicated antibodies are shown. Arrows indicate mono- or di-ubiquitinated phospho-β-catenin.

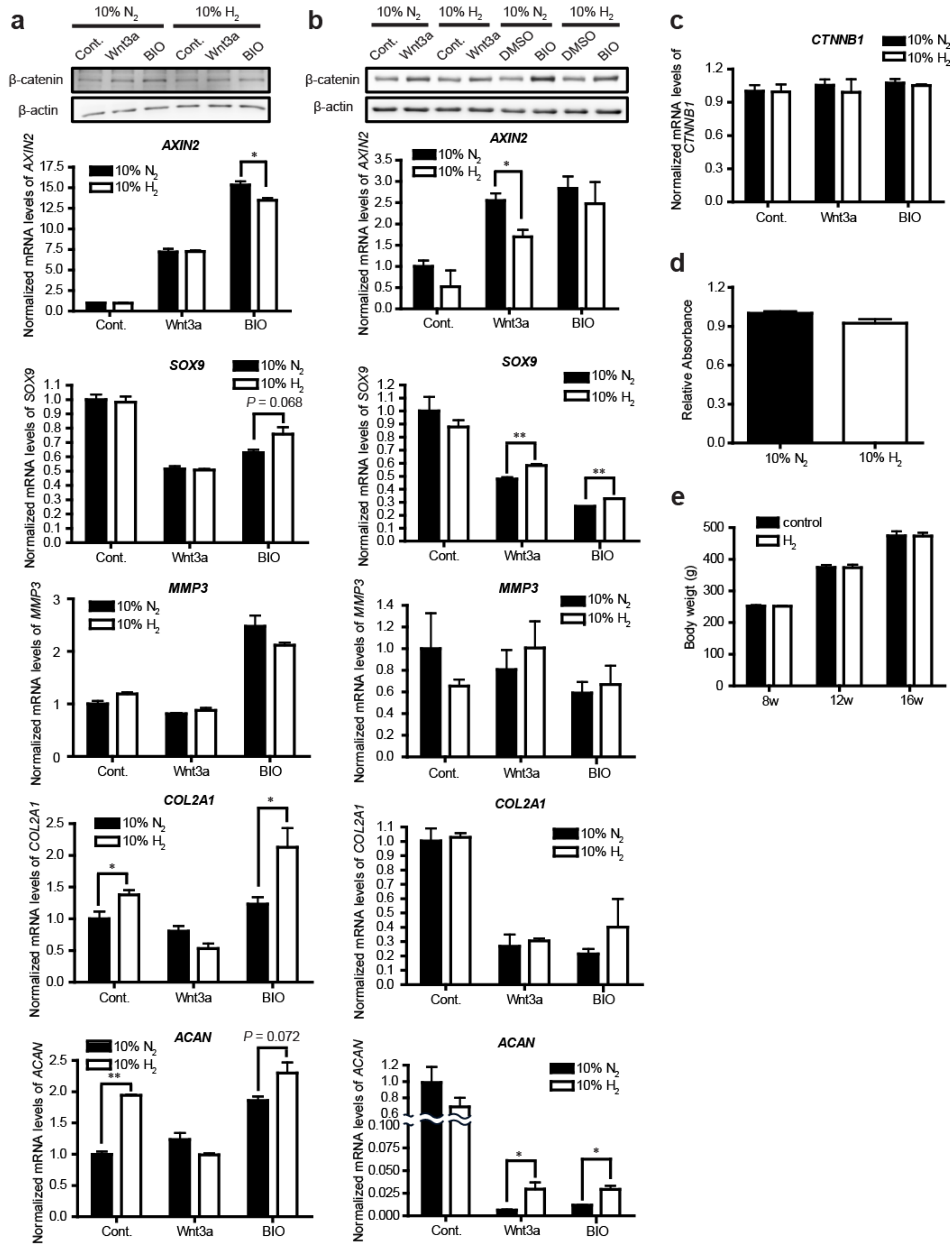

Supplemental Figure 4. H_2 inhibits Wnt/β-catenin signaling in human OAC cells.

(a) Human OAC cells (clone 2) were treated with control CM (Cont.), Wnt3a CM, or 2 μM BIO with 10% H_2

or 10% N_2 gas for 24 h. β-catenin and β-actin were detected by Western blotting. Expression of *AXIN2* (*n* = 3), *SOX9* (*n* = 3), *MMP3* (*n* = 3), *COL2A1* (*n* = 3), and *ACAN* (*n* = 3) were quantified by qRT-PCR. **P* < 0.05, ***P* < 0.01 by Student's *t*-test. Non-significant *P* values less than 0.10 are indicated above each pair.

(b) Human OAC cells (clone 3) were treated with control CM (Cont.), Wnt3a CM, 0.02% DMSO, or 2 μM BIO with 10% H_2 or 10% N_2 gas for 24 h. Western blotting and qRT-PCR were performed as in **(a)**. **P* < 0.05, ***P* < 0.01 by Student's *t*-test.

(c) Human OAC cells (clone 1) were treated with control CM (Cont.), Wnt3a CM, or 2 μM BIO with 10% H_2 or 10% N_2 gas for 24 h. Expression of *CTNNB1* was quantified by qRT-PCR (*n* = 3). No statistical difference by Student's *t*-test.

(d) ATDC5 cells were treated with 10% H_2 or 10% N_2 gas for 48 h. Proliferation of ATDC5 cells was evaluated by the BrdU cell proliferation assay (*n* = 4). No statistical difference by Student's *t*-test.

(e) Body weights of rats taking degassed water (control) or H_2 water were measured at indicated weeks of age (*n* = 6). No statistical difference by two-repeated measures ANOVA.
